# Epstein-Barr Virus Nuclear Antigen 3A Promotes Cellular Proliferation by Repression of the Cyclin-Dependent Kinase Inhibitor p21WAF1/CIP1

**DOI:** 10.1371/journal.ppat.1004415

**Published:** 2014-10-02

**Authors:** Melissa L. Tursiella, Emily R. Bowman, Keith C. Wanzeck, Robert E. Throm, Jason Liao, Junjia Zhu, Clare E. Sample

**Affiliations:** 1 Department of Microbiology and Immunology, Pennsylvania State University College of Medicine, and the Penn State Hershey Cancer Institute, Hershey, Pennsylvania, United States of America; 2 Department of Biochemistry, St. Jude Children's Research Hospital, Memphis, Tennessee, United States of America; 3 Department of Public Health Sciences, Pennsylvania State University College of Medicine, and the Penn State Hershey Cancer Institute, Hershey, Pennsylvania, United States of America; Tulane Health Sciences Center, United States of America

## Abstract

Latent infection by Epstein-Barr virus (EBV) is highly associated with the endemic form of Burkitt lymphoma (eBL), which typically limits expression of EBV proteins to EBNA-1 (Latency I). Interestingly, a subset of eBLs maintain a variant program of EBV latency - Wp-restricted latency (Wp-R) - that includes expression of the EBNA-3 proteins (3A, 3B and 3C), in addition to EBNA-1. In xenograft assays, Wp-R BL cell lines were notably more tumorigenic than their counterparts that maintain Latency I, suggesting that the additional latency-associated proteins expressed in Wp-R influence cell proliferation and/or survival. Here, we evaluated the contribution of EBNA-3A. Consistent with the enhanced tumorigenic potential of Wp-R BLs, knockdown of EBNA-3A expression resulted in abrupt cell-cycle arrest in G0/G1 that was concomitant with conversion of retinoblastoma protein (Rb) to its hypophosphorylated state, followed by a loss of Rb protein. Comparable results were seen in EBV-immortalized B lymphoblastoid cell lines (LCLs), consistent with the previous observation that EBNA-3A is essential for sustained growth of these cells. In agreement with the known ability of EBNA-3A and EBNA-3C to cooperatively repress p14^ARF^ and p16^INK4a^ expression, knockdown of EBNA-3A in LCLs resulted in rapid elevation of p14^ARF^ and p16^INK4a^. By contrast, p16^INK4a^ was not detectably expressed in Wp-R BL and the low-level expression of p14^ARF^ was unchanged by EBNA-3A knockdown. Amongst other G1/S regulatory proteins, only p21^WAF1/CIP1^, a potent inducer of G1 arrest, was upregulated following knockdown of EBNA-3A in Wp-R BL Sal cells and LCLs, coincident with hypophosphorylation and destabilization of Rb and growth arrest. Furthermore, knockdown of p21^WAF1/CIP1^ expression in Wp-R BL correlated with an increase in cellular proliferation. This novel function of EBNA-3A is distinct from the functions previously described that are shared with EBNA-3C, and likely contributes to the proliferation of Wp-R BL cells and LCLs.

## Author Summary

Epstein-Barr virus (EBV) infects over 98% of the population worldwide and is associated with a variety of human cancers. In the healthy host, the virus represses expression of its proteins to avoid detection by the immune system to enable it to remain in the body for the lifetime of its host, a situation known as latency. This downregulation was first observed in EBV-associated Burkitt lymphoma (BL), which classically express only one viral protein, EBNA-1. A subset of BL named Wp-restricted (Wp-R) BL express additional latency-associated viral proteins. Because Wp-R BL also express wild-type p53 (which normally prevents cellular proliferation), we wanted to explore the possibility that these viral proteins play a role in tumorigenesis. Indeed, we have demonstrated that Wp-R BL cells are more tumorigenic in immunocompromised mice than other BL. Here, we have investigated the role of one of these viral proteins, EBNA-3A. If we inhibit the expression of EBNA-3A, Wp-R BL cells fail to proliferate and express increased p21^WAF1/CIP1^, a cellular protein that inhibits cell proliferation. These results suggest that this previously undescribed function of EBNA-3A plays a role in the proliferation and likely contributes to tumorigenesis in Wp-R BL.

## Introduction

Epstein-Barr virus (EBV) infection of primary B cells results in sustained cellular proliferation and immortalization of infected cells *in vitro*, through the expression of the viral latency-associated genes: EBV nuclear antigens (EBNAs) 1, 2, 3A, 3B, 3C and LP, latent membrane proteins (LMPs) 1, 2A and 2B, as well as a number of small noncoding viral RNAs and miRNAs, a program of viral gene expression known as Latency III (reviewed in [1]). *In vivo*, a highly restricted program of EBV latency is ultimately established - the Latency I or Latency 0 program (little or no viral protein expression) - within infected B cells that enter the resting memory cell pool, which serves as the long-term reservoir of the virus [2]. Selective expression of the viral genome-maintenance protein EBNA-1 in Latency I is required to sustain infection during periodic proliferation of these cells for their self-regeneration; Latency I is also maintained by EBV-positive Burkitt lymphoma (BL) and BL-derived cell lines. During establishment of Latency III upon primary infection of B cells, the mRNAs encoding the six EBNAs originate initially from a common B-cell-specific *EBNA*-gene promoter, Wp, and subsequently an upstream EBNA-2-activated promoter, Cp [3]–[5]. As viral gene expression transitions to the more restricted programs of EBV latent-gene expression (e.g., Latency I), Wp and Cp become epigenetically silenced and the exclusive expression of EBNA-1 originates from the promoter Qp [6], [7].

Latent EBV infection is believed to play an etiologic role in approximately 98% of cases of endemic BL (reviewed in [8], [9]). While Latency I is maintained in the majority of EBV-positive BL, a subset accounting for as many as 15–20% of BL cases support an intermediate program of latency in which latent gene transcription originates from Wp, and is referred to as Wp-restricted (Wp-R) latency [10]. Wp-R BL tumors contain both wild-type and deleted genomes, in which all of EBNA-2 and a portion of the EBNA-LP open reading frames are deleted [10]. Furthermore, the wild-type genomes are silenced and are lost upon *in vitro* culture, while the deleted genomes persist and result in the expression of a truncated EBNA-LP in addition to EBNA-1 and the EBNA-3s [10]; LMP-1 and LMP-2 proteins, however, are not expressed, presumably due to loss of EBNA-2-mediated activation of their promoters. In addition, the deletion places *BHRF1*, a viral homologue of anti-apoptotic Bcl2 protein that is primarily expressed during lytic replication, in closer proximity to Wp, resulting in increased latency-associated BHRF1 expression [11]–[14]. Importantly, persistence of the Wp-R genomes, but not wild-type, genomes, suggests that there is selective pressure for the deleted genome, likely due to the expression of an expanded repertoire of latency-associated proteins that contribute to tumorigenesis.

Both Latency I and Wp-R BL display the hallmark chromosomal translocations that lead to MYC overexpression (reviewed in [8]). While MYC efficiently drives cellular proliferation, it also induces apoptosis through the induction of ARF expression (p19^ARF^ in mice and p14^ARF^ in humans) [15], which binds to and inhibits HDM2 resulting in stabilization of p53 [16]–[19]. Not surprisingly then, the ARF-HDM2-p53 pathway is frequently inactivated in BL tumors as well as in mouse models of BL (reviewed in [20]) [21]. By contrast (although the numbers are few), the recently identified Wp-R BL cell lines (Sal, Oku and Ava) have wild-type p53 and express p14^ARF^ [22], suggesting that the additional EBV gene products in Wp-R BL may function to overcome the p53 pathway thereby inhibiting apoptosis and/or facilitating proliferation, and indeed Wp-R BL exhibit increased apoptotic resistance relative to Latency I BL [23].

Of the EBV proteins expressed in Wp-R BLs, only EBNA-3A and EBNA-3C are known to promote proliferation. Although the EBNA-3 proteins (-3A, -3B and -3C) are presumed to have arisen from gene duplication [24]–[26], EBNA-3A and 3C are essential for naïve B cell immortalization (EBNA-3B is dispensable) [27], [28] and for the continued proliferation of the resulting lymphoblastoid cell lines (LCLs) [29]–[30], likely contributing to transcriptional regulation of cellular genes. Through their association with both common (such as CBF1 [31]–[34] or CtBP [35], [36]) and unique cellular transcription factors [37], EBNA-3A and -3C regulate cellular genes that are relevant to the pathogenesis of EBV and possibly the development of Wp-R BL [11], [22], [38]–[44]. For example, EBNA-3A and EBNA-3C cooperatively repress Bim [11], [22], [40] and the CDKN2A locus encompassing the inhibitors of G1/S progression, p14^ARF^ and the CKI p16^INK4a^ in LCLs [39], [41], [42], with the latter being the primary target of EBNA-3C during EBV-mediated immortalization of primary B lymphocytes [44].

Wp-R BL provide a unique opportunity to evaluate the functions of the EBNA-3 proteins in the absence of EBNA-2 and LMP1/2, which affect proliferation and/or survival [45]–[51], and define their contribution to Wp-R BL. Here, we demonstrate that Wp-R BL exhibit enhanced tumorigenicity relative to their counterparts that maintain Latency I. Using RNAi to knockdown expression of EBNA-3A, we determined that EBNA-3A is essential for continued proliferation of Wp-R BL cells *in vitro*, with reduction of EBNA-3A resulting in G0/G1 cell-cycle arrest, followed by apoptosis. Loss of proliferation is coincident with loss of hyperphosphorylation and subsequent depletion of Rb, suggesting that EBNA-3A may affect the expression or activity of cyclin/CDK complexes. Indeed, expression of the cyclin-dependent kinase inhibitor (CKI), p21^WAF1/CIP1^, but not p14^ARF^ or p16^INK4a^, increased following EBNA-3A knockdown in Wp-R BL, and knockdown of p21 ^WAF1/CIP1^ restored proliferation. Moreover, our findings with p21^WAF1/CIP1^ were conserved within LCLs, in which p53 is wild-type and the full repertoire of latency-associated proteins are expressed. We have, therefore, identified a novel function of EBNA-3A, distinct from those that it shares with EBNA-3C, whereby EBNA-3A regulates p21 ^WAF1/CIP1^ protein expression to maintain Rb in its inactive, hyperphosphorylated state to promote cell-cycle progression.

## Results

### Wp-R BL cells possess greater tumorigenic potential

**Figure 1:**
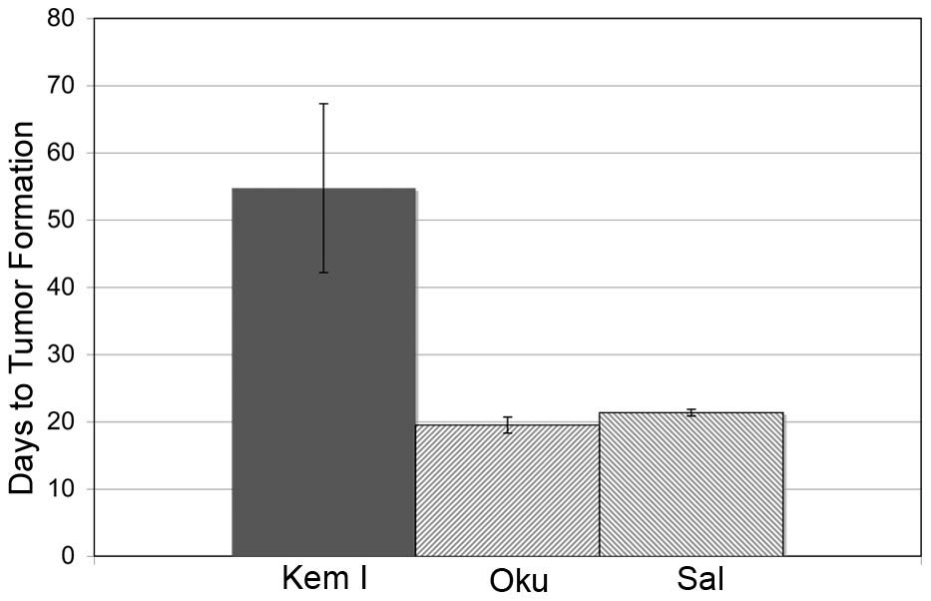
Wp-R BL cells have increased tumorigenic potential. SCID mice were injected with EBV-negative Akata BL cells (Akata^−^) on one flank and either Kem I (Latency I) or Wp-R Sal or Oku BL cells on the opposite flank. The endpoint for tumor formation was set at 2 cm in diameter. No tumors developed on flanks injected with Akata^−^ cells. The time to tumor development for Wp-R BLs (Sal or Oku) was statistically different (p<0.001 using either student's T test or Mann-Whitney U test) from Latency I (Kem I).

The significant frequency of Wp-R BLs carrying a common deletion within their endogenous EBV genomes suggests that the expanded repertoire of EBV gene products may contribute to tumorigenesis, most likely through effects on cell proliferation and/or survival. Indeed, the BHRF1 protein imparts significant resistance to apoptosis [12], [23], [52]. To begin to address this possibility, we performed xenograft assays comparing two Wp-R BL cell lines (Oku and Sal) with a standard Latency I BL cell line (Kem I) (**Fig. 1**). While tumors did not arise in mice injected with EBV-negative Akata BL cells (negative control), the average time to tumor development (2 cm in diameter) in mice injected with Latency I BL cells (Kem I) was 55 days, consistent with analysis of Latency I BLs in previous studies [53], [54]. By contrast, pea-sized tumors were detected as early as 12–14 days in mice injected with Wp-R BL cells, that reached 2 cm in diameter in an average time of 20 days, significantly sooner than the tumors derived from BL cells that maintain Latency I (p<0.001). This finding suggested that the additional EBV gene expression in Wp-R BL has a substantial influence on tumorigenic potential, at least within the context of this particular form of BL.

### EBNA-3A is essential for the proliferation of Wp-R BL cells

**Figure 2:**
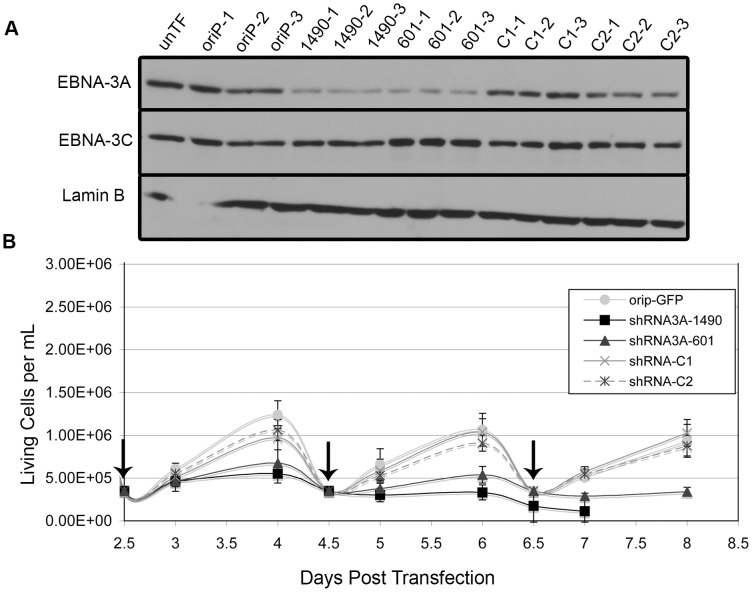
EBNA-3A is essential for proliferation of the Wp-R BL cell line Sal. Sal BL cells were transfected with empty vector (*oriP*-GFP), *oriP*-GFP encoding non-EBNA-3A-targeting control shRNA (C1 and C2), or two different EBNA-3A-specific shRNAs (1490 and 601). (A) EBNA-3A and EBNA-3C expression at 4 days post-transfection was monitored by immunoblotting; Lamin B served as a loading control. Untransfected cell lysate was also analyzed (unTF). (B) The average number of live cells per mL was analyzed beginning two days post-transfection. Cells were counted daily and reseeded every two days (as indicated by vertical arrows). Results shown are an average of two independent experiments performed in triplicate. Knockdown of EBNA-3A resulted in statistically significant effects on cell growth (shRNA3A-1490 p<0.001; shRNA3A-601 p = 0.005, both compared to the average of three control groups). In one experiment, shRNA3A-1490-containing cells could only be maintained for 7 days, resulting in data from only a single experiment performed in triplicate for the 8-day data point.

To specifically determine the potential contribution of EBNA-3A, we generated two distinct shRNAs that target different regions of the EBNA-3A mRNA to identify specific effects rather than off-target effects. Because we are unable to achieve 100% transfection efficiency, shRNAs were expressed from a vector containing a selectable marker and the EBV origin of latent DNA replication, *ori*P, which allows for stable episomal maintenance of the plasmid in EBNA-1-expressing cells. Following transfection, cells were placed in media containing G418 for the duration of the experiment to inhibit proliferation of untransfected cells (transfection efficiency was typically 75–80% as determined by flow cytometry for GFP 48 hours post-transfection). Introduction of either EBNA-3A-specific shRNA (shRNAs 1490 and 601) into the Wp-R BL cell line Sal resulted in the specific knockdown of EBNA-3A as early as 2 days post-transfection but maximal by 4 days post-transfection (**Fig. 2A**). Concomitant with EBNA-3A knockdown, cells that received an EBNA-3A-specific shRNA, but not the empty shRNA expression vector (oriP-GFP) or two control shRNAs (shRNA3A-C1 and shRNA3A-C2), ceased to expand (**Fig. 2B**). To ensure that the requirement for EBNA-3A was not specific to the Sal cell line, the experiment was repeated with a second Wp-R BL cell line, Oku, with equivalent results (**Fig. S1**). Oku cells survived transfection more poorly than Sal cells, and thus, most of our downstream analysis was performed with Sal cells. However, the loss of proliferation in Sal cells following knockdown of EBNA-3A was so rapid that very few cells could be obtained in each experiment, making downstream analysis challenging. Though not all cellular pathways could be examined in a single experiment, each experiment included verification of EBNA-3A knockdown and loss of proliferation in addition to analysis of other proteins and was repeated multiple times with both EBNA-3A targeting shRNAs to determine the mechanism by which EBNA-3A promotes the capacity of Wp-R BL cells to expand *in vitro*. Notably, we observed no effect of the EBNA-3A-targeting shRNAs when expressed in an EBV-negative BL cell line (Louckes) that we engineered to express EBNA-1 for maintenance of the *ori*P vector (**Fig. S2**). Importantly, no changes were observed in the expression of EBNA-3C (**Fig. 2A**), and knockdown of EBNA-3C in Wp-R BL cells did not result in a loss of proliferation, suggesting that EBNA-3A possesses a unique function in Wp-R BL.

### Apoptosis follows the loss of EBNA-3A-dependent cellular expansion

**Figure 3:**
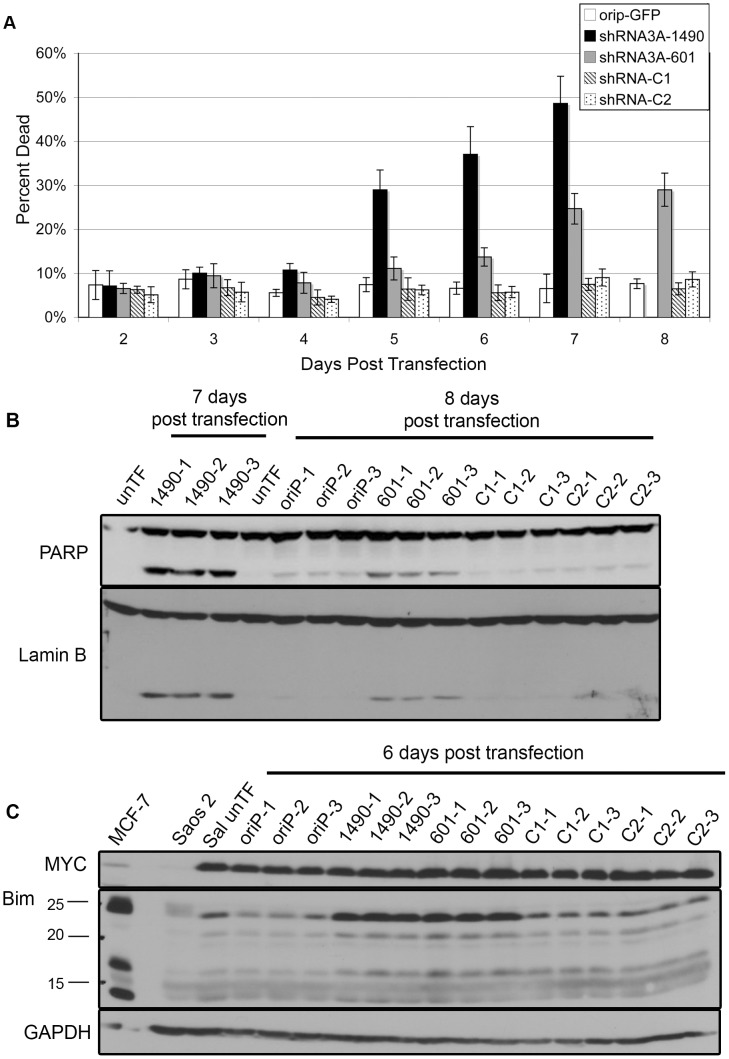
Reduced EBNA-3A expression is associated with increased expression of Bim and cell death. (A) The percentage of dead cells was measured following knockdown of EBNA-3A in two independent experiments performed in triplicate, and was determined to differ significantly between groups using Tukey's test on pairwise comparison at each time point. The controls were combined as one group for analysis. Cells expressing shRNA3A-1490 at days 3–7 were statistically different from control group at day 3 (p = 0.024) and days 4–7 (p<0.001). Those expressing shRNA3A-601 were statistically different from the control groups at day 4–8: day 4, p<0.001; day 5, p = 0.005; days 6–8, p<0.001. Cells expressing shRNA3A-1490 at days 5–7 were also statistically different from those expressing shRNA3A-601 at day 4 (p = 0.010), and days 5–7 (p<0.001). Immunoblot detection of (B) PARP and Lamin B (loading control) and (C) Bim, and GAPDH (loading control).

While inhibition of cellular expansion could be the result of increased apoptosis and/or decreased cellular proliferation, we noticed a progressive loss of cell viability associated with EBNA-3A knockdown beginning at 5 days post-transfection (**Fig. 3A**). These cells exhibited morphological changes consistent with apoptosis, and cleavage of poly-ADP-ribose polymerase (PARP) and Lamin B, both of which are substrates of the apoptotic caspase enzymes [55]–[58]. Cleavage of PARP and Lamin B was readily detectable by 6 days post–transfection, but was most prominent at 7–8 days (**Fig. 3B**), correlating with the increased loss of cell viability. Given that the proliferation of BL cells is largely driven by deregulated expression of MYC [59], [60], which can also induce apoptosis [61], we considered the possibility that these changes were mediated through alterations in the levels of MYC, though we found this not to be the case (**Fig. 3C**). Because BHRF1 was not decreased following EBNA-3A knockdown and EBNA-3A is known to repress expression of the pro-apoptotic protein Bim in LCLs [11], [22], [40], we considered that increased Bim expression could be mediating apoptosis. As shown in Figure 3C, cells expressing either EBNA-3A-specific shRNA exhibited increased levels of Bim by 6 days post-transfection, i.e., after the loss of cellular expansion, but in parallel with increased apoptosis. Thus, at least one contribution of EBNA-3A that is likely to affect tumorigenic potential in Wp-R BL is an inhibition of apoptosis mediated by its repression of Bim expression.

### EBNA-3A promotes G1/S cell-cycle progression and maintenance of Rb hyperphosphorylation independent of p14^ARF^ and p16^INK4a^ repression in Wp-R BL

**Figure 4:**
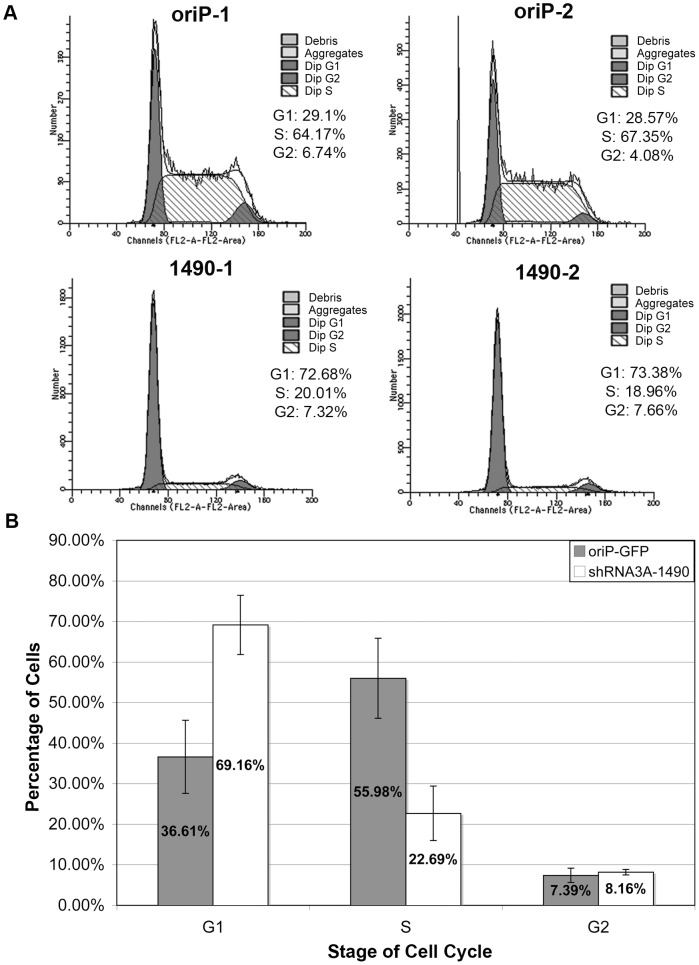
Knockdown of EBNA-3A results in G0/G1 cell-cycle arrest. (A) Cell cycle profiles of GFP-positive Sal cells 3 days post-transfection with oriP-GFP empty vector (top) or oriP-GFP-encoding shRNA3A-1490 (bottom). (B) Data from three independent experiments, each performed in duplicate, were analyzed using the two sample student T-test, and demonstrated statistically significant differences between the percentage of cells in G0/G1 (p<0.001) and S (p<0.001) phases in EBNA-3A positive (oriP-GFP) compared to knockdown cells (shRNA3A-1490).

Although reduction of EBNA-3A clearly resulted in progressive cell death, the loss of cell expansion occurred more rapidly, suggesting that the immediate effect of EBNA-3A knockdown was more likely a decrease in proliferative capacity. We therefore assessed whether knockdown of EBNA-3A caused an arrest at a specific phase of the cell cycle. Notably, as early as 3 days post-transfection, EBNA-3A-deficient cells arrested in the G0/G1 phase of the cell cycle (**Fig. 4** **and Table S1**). By contrast, the cell cycle profile of cells transfected with the empty shRNA expression vector did not change (**Fig. 4** **and Table S1**), and was similar to results obtained with expression of a control shRNA that was not EBNA-3A-specific (**Table S1**). These data suggested that EBNA-3A was likely affecting a regulator of the G1/S restriction point.

**Figure 5:**
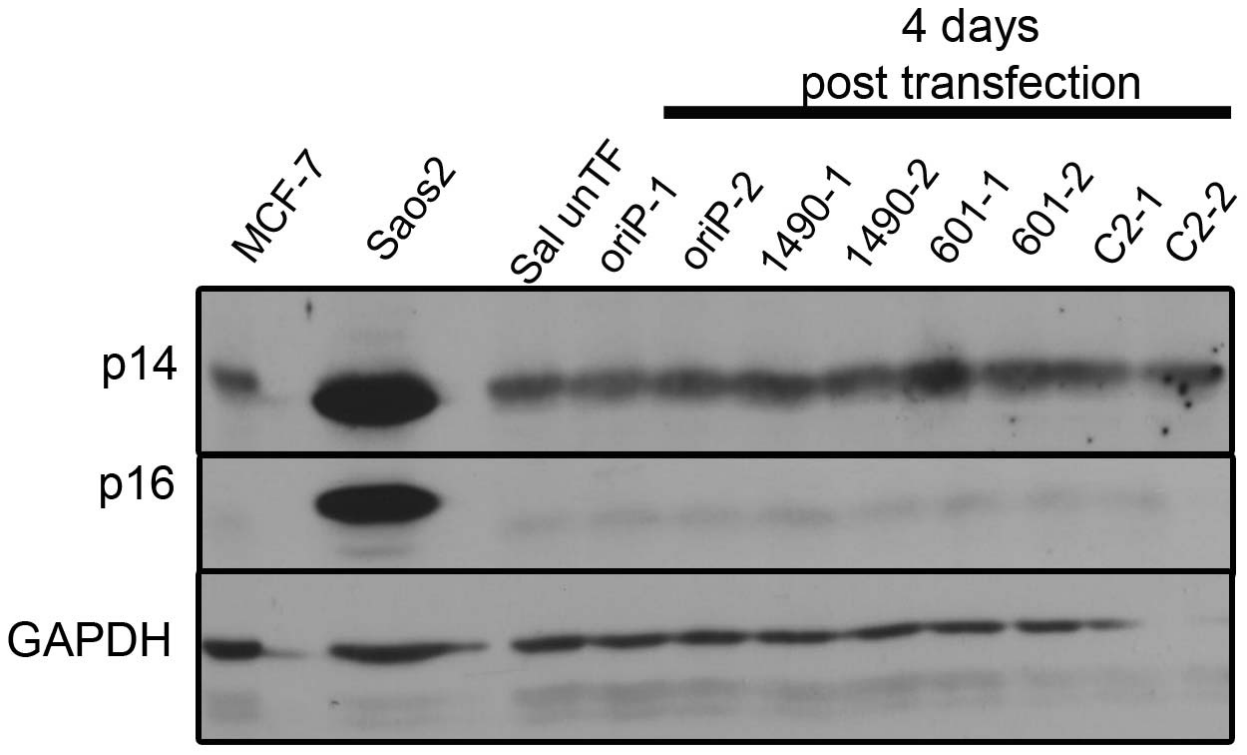
EBNA-3A does not repress p14^ARF^ or p16^INK4a^ expression in in Wp-R BL. The levels of p14^ARF^ and p16^INK4a^ in Sal BL were analyzed at 4 days post-transfection and not detected. Sal cells were transfected in duplicate with expression vectors for either EBNA-3A-specific shRNA 1490 or 601, control shRNA (C2) or empty vector (oriP). Saos2 and MCF7 serve as positive controls for detection of p14^ARF^ and p16^INK4a^; GAPDH, loading control. Knockdown of EBNA-3A was similar to that shown in Figure 2A.

**Figure 6:**
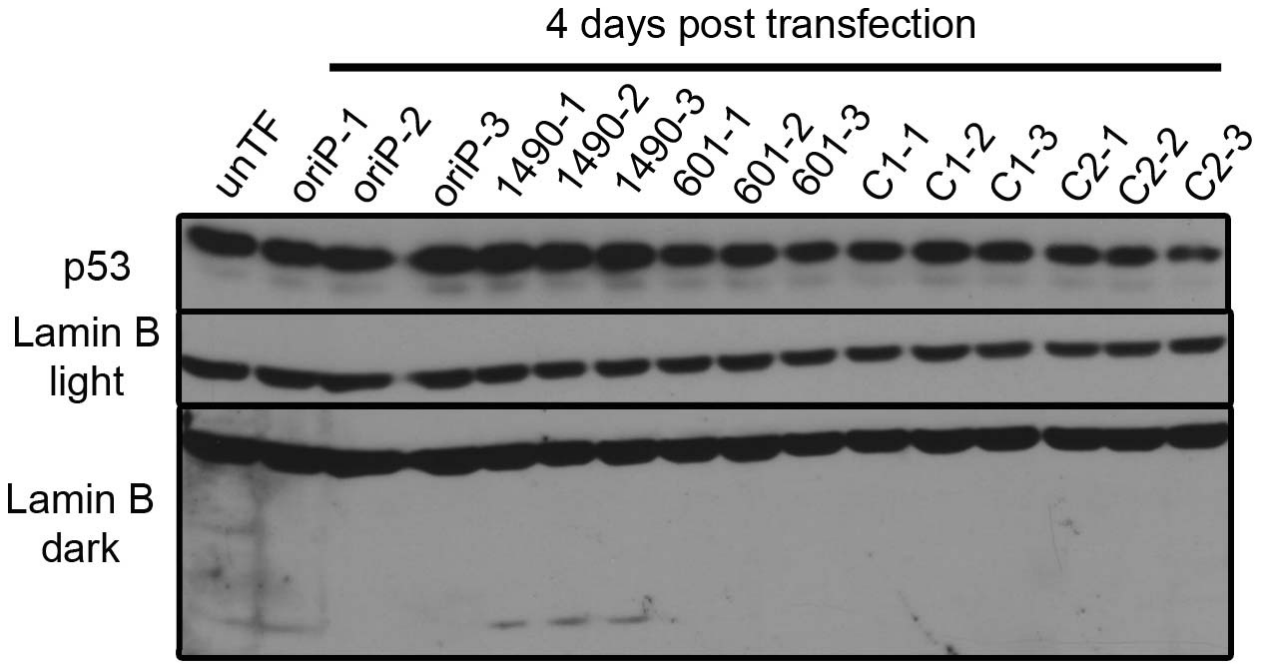
p53 expression is not modulated by EBNA-3A. Sal BL cells were transfected with the indicated shRNA expression vectors and harvested at 4 days post-transfection for immunoblot analysis of p53 and Lamin B. Designations light and dark refer to exposure time. The faster migrating Lamin B band is a cleavage product indicative of apoptosis.

Unlike many tumors, p53 is wild-type in Wp-R BL [22] and Rb is expressed and an appropriate size for wild-type protein, suggesting that it too is wild-type. Both play major roles in controlling the G1/S transition, and could be affected by EBNA-3A-enforced changes in p14^ARF^ and p16^INK4a^ expression [39], [41], [42]. Therefore, we anticipated that the proliferative advantage provided by EBNA-3A would likely be due to its ability to repress p14^ARF^ and p16^INK4a^ expression as formerly observed in LCLs. Surprisingly, levels of p14^ARF^ in Sal Wp-R BL cells were low and remained unaltered following knockdown of EBNA-3A, whereas p16^INK4a^ was undetectable regardless of EBNA-3A level (**Fig. 5**). We next analyzed the expression of p53 itself. Unlike mutant p53, wild-type p53 does not generally accumulate to high levels, yet we found that p53 was highly expressed in Wp-R BL (**Fig. 6**), suggesting the possibility that either p53 had acquired a mutation or is functionally repressed in these cell lines. To ensure that the p53 gene in Sal and Oku cells maintained within our laboratory had not accumulated mutations, we assessed the nucleotide sequence of the p53 gene within our cell stocks and found it to be wild-type [62]. Moreover, p53 was likely transcriptionally active given the appreciable expression of two downstream targets, HDM2 [63] and the p53 upregulated modulator of apoptosis (PUMA) [64] (**Fig. S3**). Thus, no changes were observed in either the levels of p53, PUMA or HDM2 that correlated with the onset of cell-cycle arrest. In most experiments, an increase in p53 was seen several days after cell cycle arrest (7–8 days), particularly upon knockdown of EBNA-3A with shRNA3A-1490, which initiated a more rapid arrest than did shRNA3A-601 (**Fig. S4**). These data suggest that changes in p53 expression likely occurred subsequent to the loss of cellular proliferation and promote the late increase in apoptosis that we observed.

**Figure 7:**
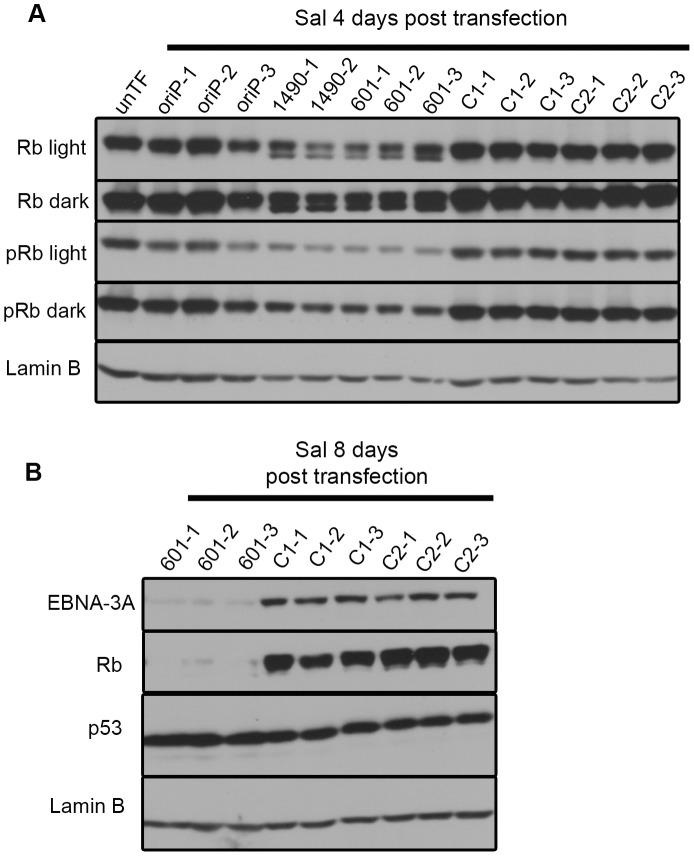
EBNA-3A supports maintenance of Rb hyperphosphorylation and stabilization in Wp-R latency. Immunoblots of transfected Sal cells harvested at (A) 4 days or (B) 8 days post-transfection were probed with a pan Rb antibody (Rb), a phospho-specific (pRb) antibody that recognizes the phosphorylated residues Ser807/811, and antibodies to detect p53 and Lamin B (loading control). Light and dark refer to film exposure time.

Because EBNA-3A did not appear to be affecting proliferation through p53, p14^ARF^ or p16^INK4a^, we considered whether EBNA-3A was affecting Rb, another key regulator of the G1/S transition. Because phosphorylation controls the activity of Rb, we determined the level of its phosphorylation by immunoblot analysis; while the slower migrating species of Rb is generally accepted to be the hyperphosphorylated species, we confirmed the identity by immunoblotting with an antibody that specifically recognizes phosphorylation of serine residues 807 and 811. Rb was highly expressed and predominantly hyperphosphorylated in both Wp-R BL cell lines (Sal, **Fig. 7A**; Oku, **Fig. S1A**). At early times following transfection with vectors encoding EBNA-3A-specific shRNAs, we observed the appearance of a prominent hypophosphorylated Rb species, as well as a corresponding decrease in hyperphosphorylated Rb (**Fig. 7A** **and** **Fig. 1A**), consistent with growth arrest in G1 (**Fig. 4** **and Table S1**). Surprisingly, we also observed an overall decrease in total Rb levels that was modest at early times post-transfection, but increased over time until little to no Rb was detected (**Fig. 7B**). Importantly, the loss of protein expression was specific to Rb, since we did not observe a decrease in other cellular or viral proteins, including p53 whose half-life is generally short (5–20 min for wild-type p53) [65].

### EBNA-3A represses expression of p21^WAF1/CIP1^ in Wp-R BL cells

Because Rb phosphorylation is controlled by the G1 cyclin-CDK complexes and their negative regulators, CKIs, we hypothesized that to promote proliferation EBNA-3A was likely influencing the expression or activity of one (or more) of these factors. We first analyzed the expression of CDKs 2, 4 and 6, and, although the expression was somewhat variable between transfected cell populations, we found no consistent changes in expression in those populations upon knockdown of EBNA-3A (**Fig. S5 A & B**). Consistent with a previous report of the Wp-R BL cell line P3HR-1 [66], cyclin D3 appeared to be the predominant cyclin D isoform in Wp-R BL, with no detectable expression of either cyclin D1 (**Fig. S5 B & C**) or D2. No consistent changes were observed, however, in either the levels of cyclin D3 or cyclin E following knockdown of EBNA-3A (**Fig. S5 A–C**).

**Figure 8:**
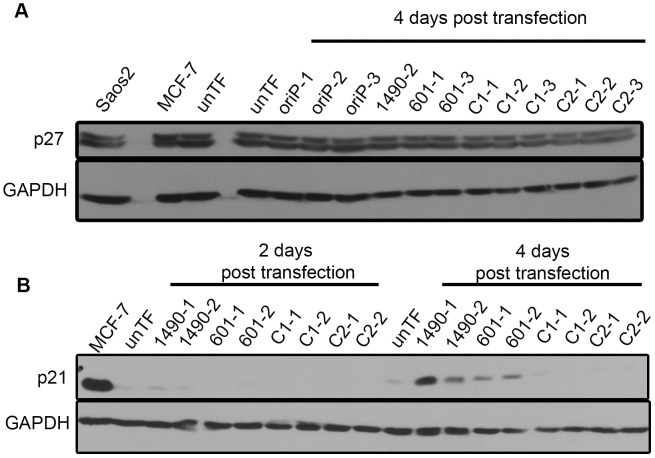
EBNA-3A represses p21^WAF1/CIP1^ expression in Wp-R BL. Cell lysates from Sal BL cells transfected with the empty shRNA expression vector (oriP-GFP) or vector encoding EBNA-3A-specific (1490 and 601), or control (C1 or C2) shRNAs were analyzed by immunoblotting to detect (A) p27 at the onset of growth arrest (4 days post-transfection); and (B) p21^WAF1/CIP1^ prior to and coincident with the onset of growth arrest at 2 and 4 days post-transfection, respectively. MCF7 and Saos2 lysates were used as positive controls; GAPDH served as a loading control. unTF, untransfected cells.

Given that EBNA-3A promoted G1/S phase progression but did not upregulate G1 cyclins or CDKs, we evaluated the expression of the CIP/KIP family of CKIs including, p21^WAF1/CIP11^, p27^KIP1^ and p57^KIP2^, whose elevated expression could mediate inactivation of cyclin D-CDK4/6 or cyclin E-CDK2 complexes (reviewed in [67]). Neither p27^KIP1^ (**Fig. 8A**) nor p57^KIP2^ (unpublished data) were upregulated following knockdown of EBNA-3A. Significantly, however, we did detect increased levels of the potent pan CKI p21^WAF1/CIP1^, which is capable of repressing both cyclin D and cyclin E-containing complexes and inducing G1 cell cycle arrest (**Fig. 8B**) [68]–[73]. The increases in p21^WAF1/CIP1^ were modest at 2 days post-transfection, but substantially elevated (4.5-fold) by 4 days post-transfection (**Fig. 8B**). Expression of p21^WAF1/CIP1^ can be upregulated by the EBV productive cycle transactivator Zta [74]–[76], but Zta protein was undetectable in Sal cells regardless of the levels of EBNA-3A (**Fig. S6**). Given that a loss of proliferation was observed between 3–4 days following expression of EBNA-3A-specific shRNAs (**Fig. 2B**), the timing of the increases in p21^WAF1/CIP1^ shown in Figure 8B is consistent with a role in the G1 cell-cycle arrest that we observed (**Fig. 4**). Together, these data suggest that the increase in p21^WAF1/CIP1^ is likely specific to the reduction in EBNA-3A expression.

**Figure 9:**
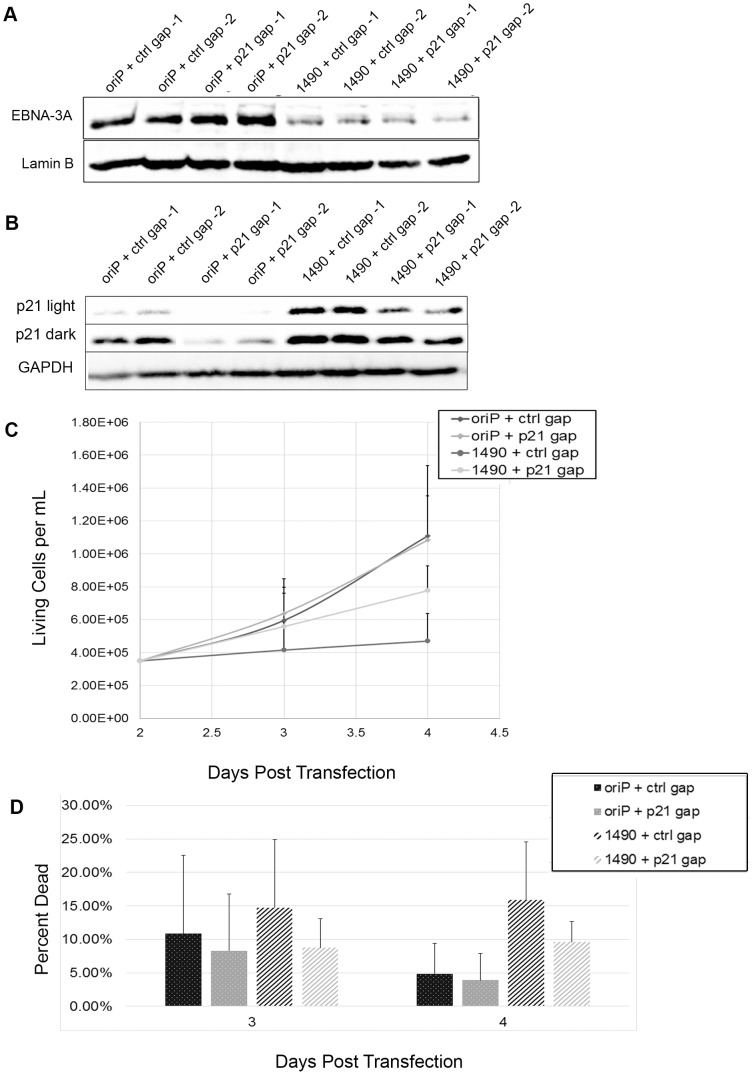
Loss of proliferation following EBNA-3A knockdown is due to p21WAF1/CIP upregulation. Sal cells were treated for 2 days with 100 nM final concentration gapmer prior to transfection. Following transfection, cells were retreated, receiving a final gapmer dose 2 days post-transfection. (A & B) Cells were harvested 4 days post-transfection and lysates were analyzed by immunoblotting for expression of (A) EBNA-3A and Lamin B or (B) p21 and Lamin B. (C & D) Cells were counted on 2, 3 and 4 days post-transfection and monitored for both live cell number (C) and viability (D).

To determine whether the elevated expression of p21^WAF1/CIP1^ was responsible for the loss of proliferation we observed, we employed LNA longRNA GapmeRs (Exiqon) that specifically target the p21^WAF1/CIP1^ transcript [77]. If the elevated p21^WAF1/CIP1^ expression was indeed mediating the loss of proliferation following EBNA-3A knockdown, reduction of p21^WAF1/CIP1^ should at least partially restore proliferation. Indeed, we find that decreasing p21^WAF1/CIP1^ expression in cells treated with EBNA-3A shRNAs resulted in maintenance of proliferation (**Fig. 9**). Growth was not restored to the level seen in control cells, likely due to the fact that the gapmers did not decrease p21^WAF1/CIP1^ to the levels seen in control cells.

### Repression of p21^WAF1/CIP1^ and stabilization of hyperphosphorylated Rb are conserved in LCLs

**Figure 10:**
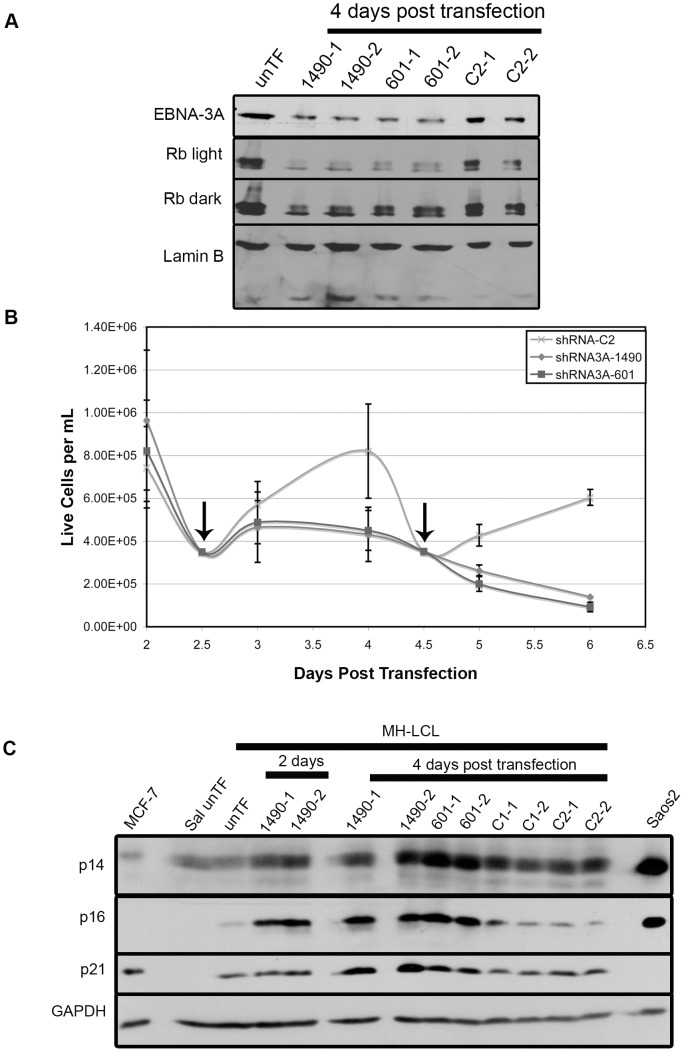
EBNA-3A support of Rb hyperphosphorylation and repression of p21^WAF1/CIP1^ are conserved in LCLs. MH-LCLs were transfected with shRNA expression vector encoding EBNA-3A-specific (1490 and 601) or control C2 shRNAs. (A) Lysates were analyzed by immunoblotting for EBNA-3A and Rb. Lamin B, loading control. Untransfected cells (unTF) served as a negative control. (B) Cell expansion was measured by counting cells in the presence of Trypan blue dye following transfection with the vector encoding the indicated shRNAs. Data shown are from 5 independent transfection experiments, each performed in duplicate. However, due to the lower rate of proliferation of LCLs and the need for removal of aliquots for protein analysis, cells were not able to be counted daily in all samples. Therefore, the number of transfected-cell samples varied at given time points: For shRNA C2, n = 8 days 2–4 and n = 4 days 4.5–6 post-transfection; for both shRNA3As 1490 and 601, n = 10 2, 2.5 and 4 days, n = 8 3 days, and n = 4 4.5–6 days post-transfection. Data shown is the mean live cell concentration with standard deviation. (C) Immunoblot detection of p14^ARF^, p16^INK4a^ and p21^WAF1/CIP1^; GAPDH, loading control.

EBNA-3A is required for the EBV-driven proliferation of LCLs [30], which is attributed to its ability to repress p14^ARF^ and p16^INK4a^ expression in cooperation with EBNA-3C [41], [42]. To further evaluate the significance of EBNA-3A-mediated repression of p21^WAF1/CIP1^, we asked whether EBNA-3A also regulated p21^WAF1/CIP1^ expression in LCLs. Knockdown of EBNA-3A in LCLs resulted in a loss of proliferation by 3 days post-transfection, which correlated with diminished hyperphosphorylated Rb and reduced total Rb levels (**Fig. 10** **A & B**) while p53 expression remained unchanged at the onset of arrest (**Fig. S7**). These findings are essentially the same as our findings in Wp-R BLs. We also observed an increase in expression of p14^ARF^ and p16^INK4a^, as described by others (**Fig. 10C**) [41], [42]. Both the loss of proliferation and the derepression of p14^ARF^ and p16^INK4a^ occurred more rapidly than previously observed in cell lines expressing a conditionally active EBNA-3A [41], [42], suggesting that the tagged EBNA-3A protein is not rapidly inactivated or possesses some function within the cytoplasm. Notably, we also observed an increase in p21^WAF1/CIP1^ similar to that we observed in Wp-R BLs (3-fold). Together, these data demonstrate that the unique ability of EBNA-3A to regulate p21^WAF1/CIP1^ is conserved in LCLs, suggesting that it is likely to be an important function of EBNA-3A.

## Discussion

Recent studies that utilized a dominant-negative EBNA-1 to induce the loss of the EBV episome have expanded our understanding of the relative contributions of EBV to Wp-R and Latency I BL [78]. Although EBV provides only minor degrees of apoptotic resistance to Latency I BL cell lines, it provides both apoptotic resistance as well as a proliferative advantage to Wp-R BL cell lines. Given that MYC is described as the critical driving force behind BL proliferation [79], these results were initially surprising. However, p53 and Rb pathways are frequently inactivated in Latency I BL (reviewed in [20]) as they are in many tumors, and mutation of p53 or p14^ARF^ accelerates tumorigenesis in a mouse model of BL [21]. Although the number examined is small, Wp-R BL cell lines contain wild-type p53 [22] that is highly expressed. Our studies indicate that Rb is also likely wild-type given that it was readily detected in a hyperphosphorylated state with wild-type mass and that a change to hypophosphorylated Rb was associated with G1/S arrest. Despite the presence of these negative regulators of the cell cycle, we demonstrate that Wp-R BL cells exhibit increased tumorigenicity over Latency I BL cells in xenograft assays, though similar rates of proliferation are observed *in vitro* (**Fig. 1**). Thus, EBV's major role in Wp-R BL is likely to be to circumvent the functional p53 and Rb pathways, and EBNA-3A likely plays a role in this process. Although BHRF1 undoubtedly plays a major role in blocking apoptosis, EBNA-3A contributes in part to apoptotic resistance but its major role is likely promoting the EBV-driven proliferation of Wp-R BL cell lines, through repression of Bim and p21^WAF1/CIP1^, respectively.

Apoptotic resistance in Wp-R BL is largely attributed to BHRF1 because, although EBNA-3A and EBNA-3C cooperatively repress Bim [11], [22], [40], the EBNA-3 proteins do not affect apoptotic resistance when expressed exogenously in a Latency I BL cell line [12]. However, the Bim promoter is generally epigenetically silenced in Latency I BL cell lines [80], [81]. Despite the presence of BHRF1, the abrupt G1 cell cycle arrest that occurred after EBNA-3A knockdown in Wp-R BL was followed by a gradual increase in apoptosis, paralleled by increases in Bim. In mouse models of BL, reduction of Bim facilitates tumor formation [82], and thus, EBV likely offers a growth advantage by countering apoptosis. Bim can be indirectly activated by p53 [82], and indeed, we observed an increase in p53 at late times post-knockdown of EBNA-3A. Our data demonstrate that EBNA-3A does indeed contribute to apoptotic resistance in Wp-R BL cell lines by decreasing levels of Bim, suggesting that BHRF1 alone may be insufficient to prevent apoptosis in all circumstances.

Although EBNA-3A does repress apoptosis in Wp-R BL, the immediate effect of knockdown of EBNA-3A was G1 cell cycle arrest, suggesting that EBNA-3A modulated the expression or activity of one or more G1/S regulatory factors. While p53 was increased at late times after knockdown of EBNA-3A, we did not see an increase in p53 coincident with cell cycle arrest, nor did we observe changes in the p53-regulated proteins, PUMA and HDM2, suggesting that EBNA-3A may not target the expression or activity of p53 directly to mediate its effects on proliferation. Although only low levels of p14^ARF^ were expressed in Wp-R BL cells, the level did not change following EBNA-3A knockdown. This result is consistent with the lack of change in p53 expression at early times post EBNA-3A knockdown because p14^ARF^ stabilizes p53 [15]–[17], [19]. Instead, we found that loss of EBNA-3A was associated with a conversion of Rb from a hyperphosphorylated inactive protein to a hypophosphorylated active species, ultimately culminating in a loss of Rb. Thus, EBV appears to stabilize Rb in an inactive hyperphosphorylated state rather than degrading it as has been proposed [83]. Although changes in phosphorylation of Rb could be mediated through any of the G1/S cyclins, CDKs and CKIs, p16^INK4A^ is known to be coordinately repressed in LCLs by EBNA-3A and EBNA-3C [41], [42], [44], and indeed, we do see increases in p16^INK4A^ in LCLs. In Wp-R BL, however, p16^INK4A^ was undetectable irrespective of EBNA-3A expression, suggesting that p16^INK4a^ is likely to be irreversible epigenetic silenced as is often observed in Latency I BL [84]. Therefore, the changes in Rb phosphorylation and total Rb protein levels that occur in Wp-R BL following knockdown of EBNA-3A must be independent of p16^INK4a^.

EBV infection results in elevated expression of various cyclins and CDKs, which promote increased Rb phosphorylation [85]–[87]. EBNA-2 orchestrates these changes likely through upregulation of MYC. Although EBNA-2 is not expressed in Wp-R BL, the translocation of MYC, which results in overexpression, likely results in similar changes in cell cycle regulatory proteins, and indeed, the G1/S cyclins and CDKs that we examined were largely expressed at similar levels in LCLs and Wp-R BLs. Despite analysis of a large panel, loss of EBNA-3A resulted in no significant changes in any of the G1 cyclins and CDKs evaluated. If EBNA-3A were instead repressing the expression of one of the CKIs, Rb phosphorylation would be reduced following EBNA-3A knockdown, inhibiting passage through the G1/S transition. Indeed, p21^WAF1/CIP1^, but not p27 (**Fig. 8**) or p57, was increased in Wp-R BL coincident with G1 growth arrest.

Increased p21^WAF1/CIP1^ expression is likely responsible for not only the hypophosphorylation of Rb associated with loss of EBNA-3A, but also the eventual decrease in total Rb protein. In colon carcinoma cell lines, similar decreases in Rb levels are elicited by exogenous expression of p21^WAF1/CIP1^, or by induction of p21^WAF1/CIP1^ by DNA-damaging agents in wild-type cells, but not in those where the gene is deleted or expression is knocked down [88]. Similar experiments have demonstrated that exogenous p16^INK4A^ mediates only hypophosphorylation of Rb with no changes in total levels of Rb protein [88]. Furthermore, reduction of EBNA-3C in LCLs results in increased p16^INK4A^ and hypophosphorylation of Rb, but no change in levels of Rb protein [29], [41]. It has been suggested that hypophosphorylated Rb is less stable than hyperphosphorylated Rb due to its preferential interaction with HDM2 and subsequent targeting to the proteasome, though how p21^WAF1/CIP^ might mediate this effect is not clear [89], [90]. Though in most cases, loss of Rb is associated with cellular proliferation, Rb is dispensable for prolonged arrest in some cases where p21^WAF1/CIP1^ is expressed, [91]. Regardless, knockdown of EBNA-3A results in cell cycle arrest coincident with Rb dephosphorylation and is likely followed by an irreversible commitment to apoptosis, suggesting that the loss of Rb may be mediated by increased caspase activity [92].

The fact that p21^WAF1/CIP1^ expression was elevated following RNAi-mediated knockdown of EBNA-3A in more than one LCL, coinciding with the onset of arrest, suggested that our finding had more global significance. While p21^WAF1/CIP1^ is best known for its potent ability to trigger growth arrest through the inhibition of cyclin/CDK-mediated Rb phosphorylation, p21^WAF1/CIP1^ also plays an important role in the activation of cyclin D-CDK4/6 complexes by promoting their assembly and nuclear translocation [68]–[72], [93]–[95]. Therefore, low levels of p21^WAF1/CIP1^, such as those seen in Wp-R BL and LCLs, may be advantageous, and EBNA-3A may promote proliferation in Wp-R BL through the fine-tuning of p21^WAF1/CIP1^ expression. Whether it does so in LCLs is complicated by the fact that EBNA-3A also represses p14^ARF^ and p16^INK4A^ [41], [42], and the requirement for not only EBNA-3A, but also EBNA-3C (and EBNA-2) for cellular proliferation [28], [96]–[98]. In LCLs, the major function of EBNA-3C is believed to be repression of p16^INK4a^, because the loss of EBNA-3C can be fully compensated for by shRNA-mediated knockdown of both p14^ARF^ and p16^INK4a^ [41], and EBNA-3C is not required for immortalization of p16^INK4a^-null B cells (i.e., into LCLs) [44]. Importantly, in our hands and previous studies from Maruo and colleagues, loss of EBNA-3C does not affect p21^WAF1/CIP1^ expression in LCLs [29], [62]. These data are consistent with the fact that EBNA-3C is not essential for proliferation in Wp-R BLs [62], which express minimal p14^ARF^ and lack p16^INK4a^. Conversely, the loss of EBNA-3A in LCLs is not totally compensated for by knockdown of p14^ARF^ and p16^INK4a^ [41], and EBNA-3A is required for proliferation of Wp-R BL despite the lack of p16^INK4a^ and minimal p14^ARF^, suggesting that we have discovered a novel and important function of EBNA-3A. Thus far, a majority of the pertinent functions uncovered for EBNA-3A are redundant with those described for EBNA-3C, whereas the ability to repress p21^WAF1/CIP1^ is unique to EBNA-3A. EBV inhibits the induction of p21^WAF1/CIP1^ protein expression following cisplatin treatment of LCLs despite increases in p21^WAF1/CIP1^ mRNA [99], [100], but the mechanism of regulation of p21^WAF1/CIP1^ may differ in the presence of cisplatin and/or the full complement of EBV latency-associated genes. Because EBNA-3A is a transcriptional regulator, it is probable that the mechanism involves transcriptional regulation, either directly or indirectly at the level of the of the p21^WAF1/CIP1^ gene.

The appreciable expression of both p53 and Rb in both Wp-R BL cells and LCLs suggest that, at least in these cell types, EBV circumvents these cell cycle regulatory proteins by modifying their activity rather than their expression as previously proposed [83], [101]. In Wp-R BL, BHRF1, EBNA-3A and -3C are likely all involved in preventing p53-mediated apoptosis to some degree. However, repression of p21^WAF1/CIP1^ is a novel and essential function of EBNA-3A, not shared with EBNA-3C, which prevents p53 and Rb-mediated cell-cycle arrest. Notably, this function is conserved in LCLs, and likely contributes to their establishment and continued proliferation. It therefore follows that this EBNA-3A function is likely critical in the establishment of persistent latent infection *in vivo*, in which the EBNA-3A-containing Latency III program is responsible for driving the expansion of newly infected B cells. Furthermore, the anti-apoptotic functions of EBNA-3A, -3C and BHRF1, combined with the ability of EBNA-3A to repress p21^WAF1/CIP1^, mediate the essential anti-apoptotic and pro-proliferative effects provided by EBV in Wp-R BL cells [78] and likely contribute to the development of Wp-R BL.

## Materials and Methods

### Ethics statement

All animal experiments were conducted in accordance with the recommendations in the Guide for the Care and Use of Laboratory Animals of the National Institutes of Health. All experimental protocols were approved by St. Jude Children's Research Hospital Institutional Animal Care and Use Committee (Protocol # 437).

### Cell culture

Kem I, Oku and Sal are human EBV-positive BL cell lines that maintain Latency I (Kem I) or Wp-R latency (Oku and Sal). Akata^−^ is an EBV-negative clone derived from the EBV-positive Akata BL cell line. Louckes-EBNA-1 is an EBV-negative BL cell line that contains the episomal pCEP4 plasmid that expresses EBNA-1 and is maintained in Hygromycin (100 µg/mL). MH-LCL is an EBV-immortalized B lymphoblastoid cell line. All B-cell lines were grown in RPMI 1640 (HyClone, Thermo Scientific Waltham MA) supplemented with either 10% fetal bovine serum (FBS) (BL cell lines) or 15% FBS (MH-LCLs). For transfection, cells were grown in roller bottles and used while in log phase of growth (0.35–1.2×10^6^ cells/mL). MCF-7, a human breast adenocarcinoma-derived cell line, was maintained in EMEM (Lonza Walkersville, MD) supplemented with 10% FBS and 0.01 mg/mL bovine insulin (Sigma #I1882). Saos2, a human osteosarcoma-derived cell line, was maintained in DMEM (Lonza) containing 10% FBS. All cells were grown at 37°C in a 5% CO_2_ atmosphere.

### Xenograft assay

NOD.CB17-Prkdc^scid^/J mice (Jackson Laboratory, Bar Harbor, Maine) were housed in pathogen-free conditions and used to test the tumorigenicity of BL cell lines. Immunodeficient mice were injected subcutaneously in one flank with 1×10^7^ EBV-negative Akata cells, and in the other flank with Kem I cells (n = 12) or Wp-R BL cells Oku (n = 16) or Sal (n = 14) and monitored until tumors reached 2 cm, at which time mice were sacrificed. The data were analyzed using student's T test as well as by Mann-Whitney U test, both of which yielded equivalent results, determining that the differences in time to tumor formation between Kem I and Oku or Sal were highly significant (p<0.001).

### Plasmids

Two shRNAs were designed to target different sites within the EBNA-3A mRNA: shRNA3A-601 (GGTACGAAGAGAAAGCGGGTAAGCTTACCCGCTTTCTCTTCGTACCCTTTTTG) targeting nucleotides 181–200, and shRNA3A-1490 (GGTCGTGCGTATGGGATAGAAAGC TTTCTATCCCATACGCACGACCCTTTTTG) targeting nucleotides 981–1001. A BLAST search was conducted to verify that the shRNAs would not target other viral or cellular sequences. Control, non-EBNA-3A-targeting shRNAs Control-1 (C1) (GACTTCTGAATGAGACAA CATCGAAATGTTGTCTCATTCAGAAGTC) and Control-2 (C2) (CACCGGACTACCGACGAAG GAACGAGAGTTCCTTCGTCGGTAGTCC) were also generated. Each pair of deoxyoligonucleotides was cloned into pBSU6 (generously provided by Dr. Yang Shi, Harvard Medical School [102]) under control of the cellular U6 promoter, and tested for the ability to knockdown EBNA-3A expression in HEK-293T transient transfection with these shRNA expression vectors. A *Bam*HI fragment containing the U6 promoter-shRNA cassette was then removed and cloned into the *Xba*I site of the vector *oriP*-GFP (gift of Jeffery Vieira, University of Washington) and confirmed by DNA sequence analysis. The *oriP*-GFP vector contains a neomycin-resistance (neoR) gene, GFP (both under the control of constitutive promoters) and the EBV origin of latent DNA replication, *oriP*, allowing for stable, episomal maintenance of the plasmid by EBNA-1 in EBV-positive cells.

### Transfection

Sal cells were transfected by Amaxa nucleofection (Lonza) using program G-16 and solution V (Lonza). Prior to transfection, cells were seeded at 3.5×10^5^ cells/mL and maintained in roller bottles for 48 hours; after which 5.0×10^6^ cells were transfected with 5 µg of plasmid DNA and plated in a 12-well plate with 0.5 mL conditioned media and 0.5 mL fresh RPMI growth media per well. Cells were fed with 1.5 mL of fresh growth media at 24 hours post-transfection. At 48 hours post-transfection, GFP expression was monitored by fluorescence microscopy, and cells seeded at 3.5×10^5^ cells/mL with 1∶1 mixture of fresh and conditioned RPMI growth media from the parental cell line, and thereafter re-seeded every other day as indicated using a 1∶1 mixture of fresh and conditioned medium containing G418 at 600 µg/mL. Cell viability was assessed by Trypan blue dye exclusion. Oku cells were transfected as described above for Sal. Louckes-EBNA-1 were also transfected as described above, however in addition to G418, they were also given Hygromycin (100 µg/mL). LCLs were transfected by Amaxa nucleofection (Lonza) using Solution V and either program O-17 or G-16 (with the latter resulting in higher transfection efficiency with less cell death, but no differences in experimental results) and were handled basically as described above. Cells were initially expanded in roller bottles, and a total of 1×10^7^ cells were used per transfection. Following nucleofection, the cells were plated at 3.5–4.0×10^5^ cells/mL in a 6-well plate containing 1.75 mL fresh RPMI containing 15% FBS and 1.25 mL of conditioned media from the parental cell line. Beginning two days post-transfection, G418 was included in the growth medium at 600 µg/mL.

### Akata induction

EBV-positive Akata cells were seeded at 5×10^5^ cells/mL and treated with 100 µg/ml of human anti-IgG antibody (ICN/Cappel) per mL of culture. Cells were induced for 2 days and then harvested.

### Gapmer gymnosis

LNA longRNA GapmeRs were ordered from Exiqon. The control gapmer (ACCagggcgtatctctccATA) [103] and p21-specific gapmer (TCCgcgcccagCTCC) [77] were administered to cells via gymnosis. The uppercase letters indicate the LNA while the lower case letters indicate phosphorothioated bases. On Day 0, the respective gapmer was added to the media for a final concentration of 100 nM. Cells were transfected, (as described above) on Day 2 and gapmer was readministered. On Day 4 (2 days post-transfection), cells were treated as described above for normal Sal transfection, with the addition of another dose of gapmer to the same final concentration.

### Immunoblotting

Cells were harvested by centrifugation, washed twice with phosphate buffered saline (PBS) and lysed in a derivative of Laemmli sample buffer [104] (125 mM Tris-HCl pH 6.8, 4% SDS, 25% glycerol, 0.01% Bromophenol blue and 10% beta-mercaptoethanol) and sonicated. Proteins were separated by SDS-PAGE, using the lysate equivalent of 1×10^6^ B cells (BL or LCL), 2.5×10^5^ MCF-7 cells, or 0.5–1.0×10^6^ Saos2 cells loaded per lane, and then transferred onto PVDF membranes. Membranes were blocked in 5% milk in Tris-buffered saline containing 0.1% Tween 20 (TBST), prior to incubation with primary antibody. The following primary antibodies were used for immunoblotting: anti-EBNA-3A and -3C sheep serum (Exalpha); Bim (BD Biosciences, Franklin Lakes, NJ); MYC (9E10 hybridoma supernatant); CDK4, CDK6, p21^WAF1/CIP1^ (12D1), PUMA, Rb (4H1), pRB (recognizing phosphorylation of S807/811), Cyclin D1 and Cyclin D3 (all from Cell Signaling Danvers, MA); Cyclin E (BD Pharmingen, Franklin Lakes, NJ); PARP (Roche, Indianapolis, IN); p16^INK4A^ (mAb4133 cloneD25; Millipore, Billerica, MA); CDK2, p14^ARF^ (C-18), p27^KIP1^ (C19), p53 (DO-1), HDM2 (SMP14), Zta (BZ1), GAPDH (FL335), and Lamin B (M20) (all from Santa Cruz Biotechnology, Santa Cruz, CA). Immunoblots were developed with ECL+, ECL prime (GE Amersham, Piscataway, NJ) or SuperSignal West Pico (Pierce, Rockford, IL). Immunoblots for p21 were quantified using a Bio-Rad ChemiDocMP imaging system and Image Lab image copture and analysis software.

### Propidium iodide staining and cell cycle analysis

Transfected cells (1.0×10^6^ cells) were harvested for propidium iodide (PI) staining at 3, 4 or 5 days post-transfection, and, in some experiments, fixed in 1% paraformaldehyde in PBS for 1 hour at 4°C to maintain endogenous GFP. Cells were subsequently centrifuged at 300× *g* at 4°C, washed once in PBS and resuspended in 70% ethanol. After 2 hours, cells were centrifuged at 300× *g*, rehydrated in PBS, centrifuged and resuspended in 705 µL of PBS containing 40 µg/mL PI (Invitrogen, Carlsbad, California) and 200 µg/mL RNase A (Invitrogen), followed by incubation in the dark for 30 minutes at room temperature. Following PI staining, cells were analyzed at a low flow rate on a FACScan Flow Cytometer (BD Biosciences, Franklin Lakes, NJ), and a minimum of 10,000 events were collected. Cell-cycle analysis was calculated based on measured DNA content of either GFP-positive cells or total cells using ModFit LT software (Verity Software House, Topsham, ME) with both methods yielding consistent results.

## Supporting Information

Figure S1 **EBNA-3A is essential for proliferation of the Wp-R BL cell line Oku.** Oku cells were transfected using the same protocol previously described for Sal but, could only be carried out to 3 days for most experiments due to poor viability post-transfection. (A) Transfected Oku cells were harvested at 3 days and lysates were assessed by immunoblotting for EBNA-3A and Rb. Lamin B served as a loading control. Trypan blue exclusion was used to count cells (B) and assess viability (C). Data shown is an average of two experiments with duplicate transfections.
(TIF)

Figure S2 **Transfection of shRNA3A does not result in death of the EBV-negative BL Louckes- EBNA-1.** Louckes-EBNA-1 cells were transfected in duplicate using the same protocol used for Sal cells. Cells were assessed daily via Trypan blue exclusion and live cell number (A) and viability (B) were analyzed. Arrows in A indicate that cells were reseeded at 3.5×10^5^ cells/mL in half conditioned media containing the appropriate drugs for selection.
(TIFF)

Figure S3 **Knockdown of EBNA-3A has no effect on p53 effectors PUMA and HDM2.** Sal BL cells were harvested at 2 or 4 days post-transfection and lysates analyzed by immunoblotting to detect (A) EBNA-3A and HDM2 (p90-active; p60-inactive forms) or (B) PUMA. Lamin B and GAPDH served as loading controls.
(TIF)

Figure S4 **Elevated p53 at late times post-transfection correlates with apoptosis rather than the onset of arrest.** Sal cells were transfected in triplicate. Due to the low density and poor viability, shRNA3A-1490 samples could not be maintained until 8 days and were harvested at 7 days. Immunoblots of EBNA-3A, p53, PARP and Lamin B are shown. Note: PARP and Lamin B immunoblots are from Figure 4 and are included here to illustrate the apoptosis occurring in parallel with p53 expression.
(TIF)

Figure S5 **EBNA-3A does not affect expression of G1/S cyclin or CDKs.** Immunoblot analysis was performed for (A) CDKs 4, 6, and cyclin E; (B) CDK2 and cyclin D3; and (C) cyclin D1 using lysates from Sal cells transfected with either empty shRNA expression vector (oriP), EBNA-3A-specific (1490 and 601) or control shRNAs (C1 and C2). GAPDH served as a loading control. Representative time points post-transfection are shown, but expression of all proteins was analyzed at 2, 4, and 6 days post-transfection, with no consistent difference between samples, regardless of the level of EBNA-3A.
(TIFF)

Figure S6 **Increased p21 expression following EBNA-3A knockdown is not due to Z expression and lytic reactivation.** Sal cells were transfected as previously described and harvested at 4 or 8 days post-transfection in two independent experiments. The productive cycle of replication was induced in EBV-positive Akata cells, which serve as a positive control for Z expression. The EBV-negative BL cell line BL2 serves as a negative control. Immunoblots for Z and GAPDH are shown.
(TIF)

Figure S7 **Loss of proliferation in LCLs following EBNA-3A knockdown is not due to elevated p53 expression.** MH-LCLs were transfected as described previously, and lysates were harvested at 4 days post-transfection. Immunoblots for p53 and Lamin B are shown.
(TIF)

Table S1 **Knockdown of EBNA-3A with either shRNA results in G0/G1 cell cycle arrest while control shRNAs have no effect.** Sal cells were transfected as described previously and cell cycle analysis was performed as described for Figure 4.
(TIF)
