# Supplementary figures and images for: Epstein-Barr Virus Nuclear Antigen 3A Promotes Cellular Proliferation by Repression of the Cyclin-Dependent Kinase Inhibitor p21WAF1/CIP1
Source: PLoS Pathog. 2014 Oct 2;10(10):e1004415. doi: 10.1371/journal.ppat.1004415 (PMC4183747; doi:10.1371/journal.ppat.1004415)

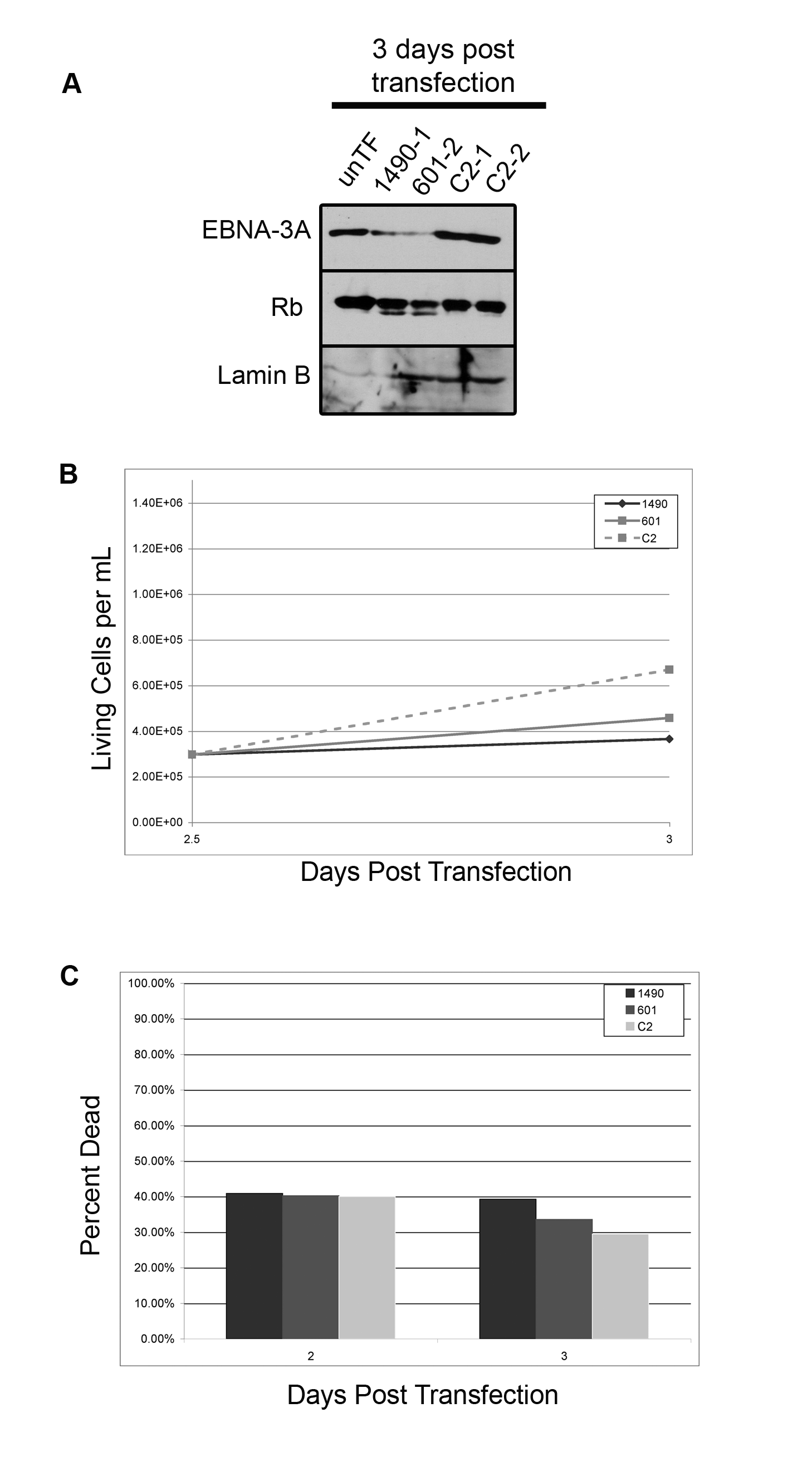

Supplement: Figure S1 — EBNA-3A is essential for proliferation of the Wp-R BL cell line Oku. Oku cells were transfected using the same protocol previously described for Sal but, could only be carried out to 3 days for most experiments due to poor viability post-transfection. (A) Transfected Oku cells were harvested at 3 days and lysates were assessed by immunoblotting for EBNA-3A and Rb. Lamin B served as a loading control. Trypan blue exclusion was used to count cells (B) and assess viability (C). Data shown is an average of two experiments with duplicate transfections. (TIF) [file ppat.1004415.s001.tif]

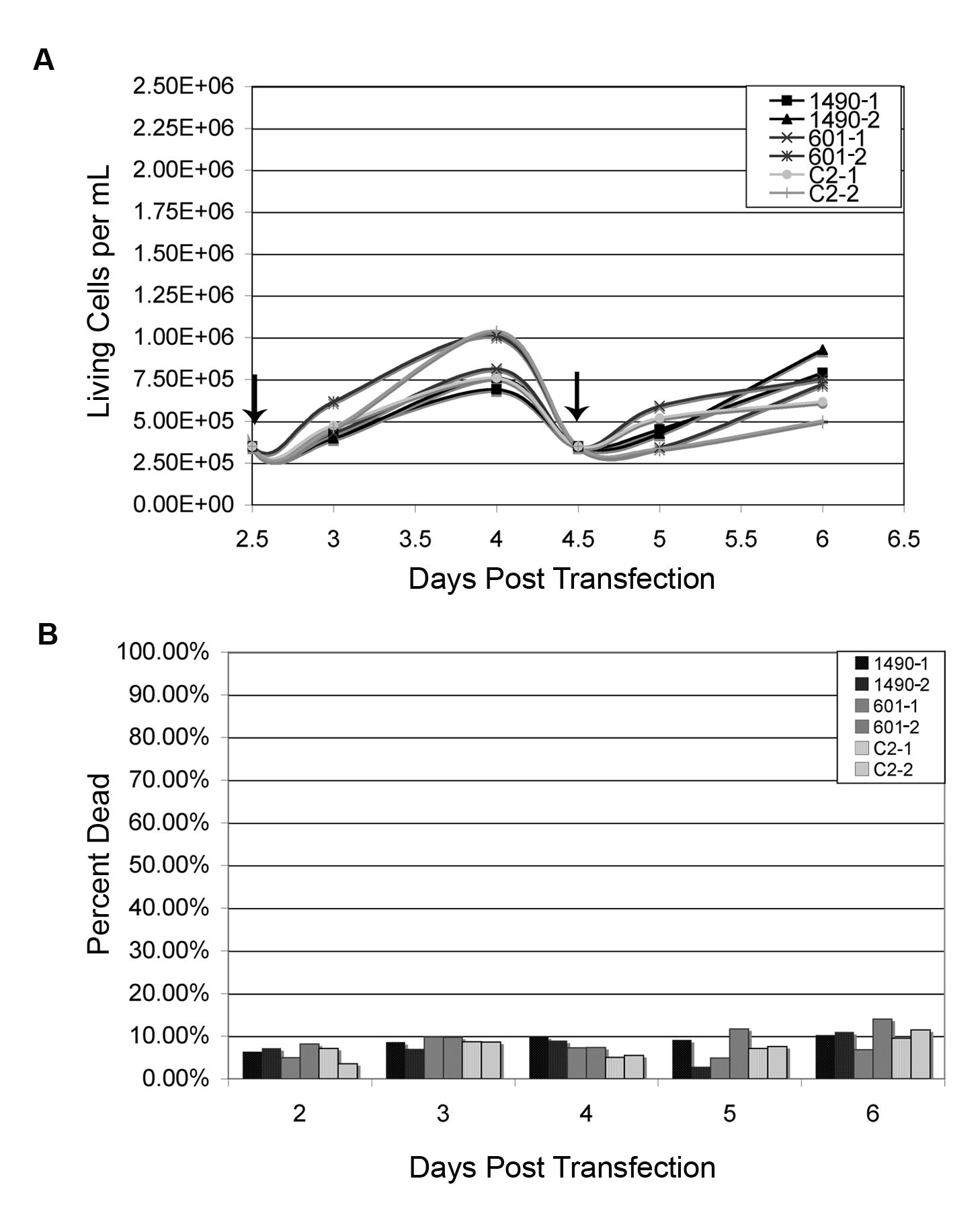

Supplement: Figure S2 — Transfection of shRNA3A does not result in death of the EBV-negative BL Louckes- EBNA-1. Louckes-EBNA-1 cells were transfected in duplicate using the same protocol used for Sal cells. Cells were assessed daily via Trypan blue exclusion and live cell number (A) and viability (B) were analyzed. Arrows in A indicate that cells were reseeded at 3.5×10^5^ cells/mL in half conditioned media containing the appropriate drugs for selection. (TIFF) [file ppat.1004415.s002.tiff]

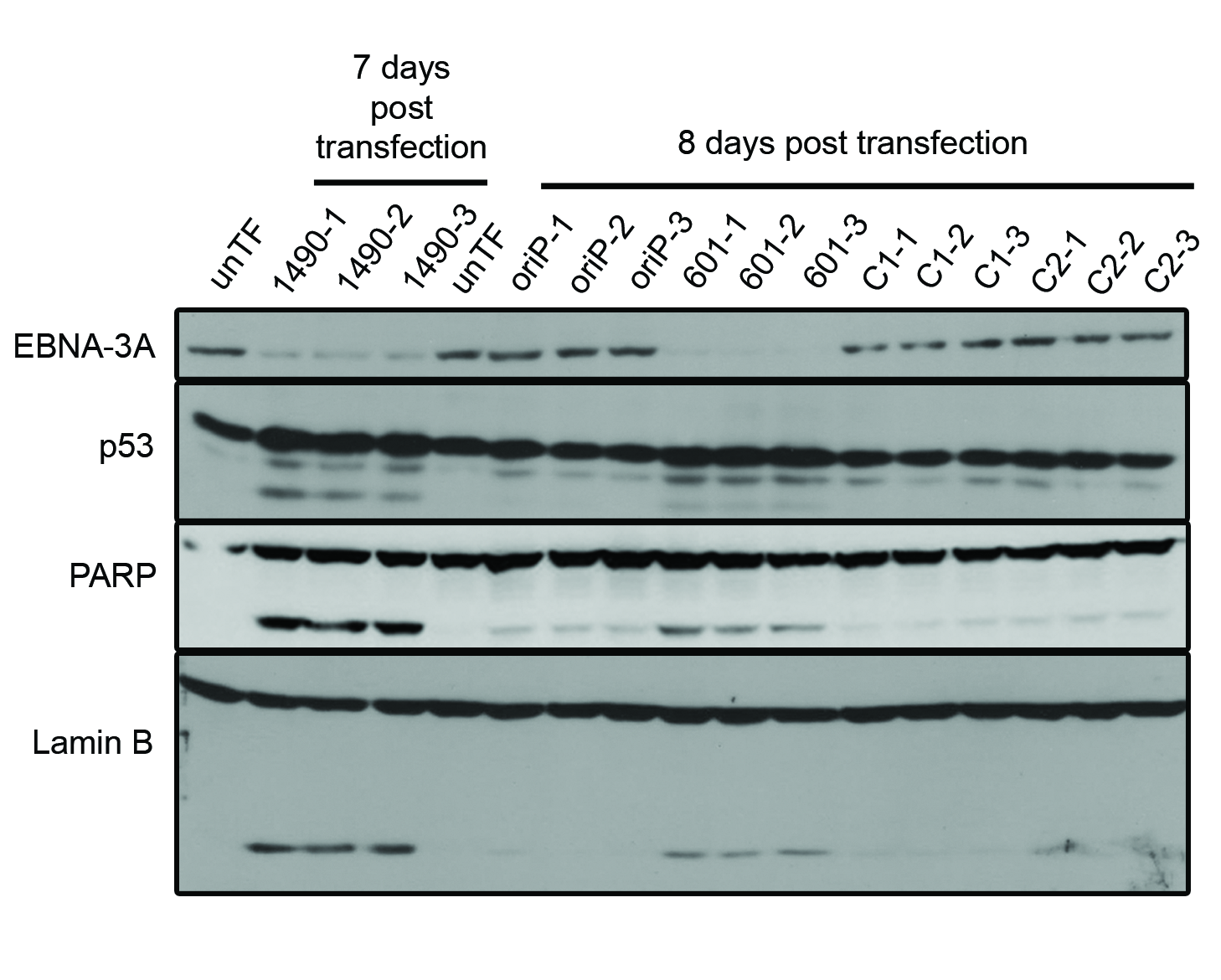

Supplement: Figure S4 — Elevated p53 at late times post-transfection correlates with apoptosis rather than the onset of arrest. Sal cells were transfected in triplicate. Due to the low density and poor viability, shRNA3A-1490 samples could not be maintained until 8 days and were harvested at 7 days. Immunoblots of EBNA-3A, p53, PARP and Lamin B are shown. Note: PARP and Lamin B immunoblots are from Figure 4 and are included here to illustrate the apoptosis occurring in parallel with p53 expression. (TIF) [file ppat.1004415.s004.tif]

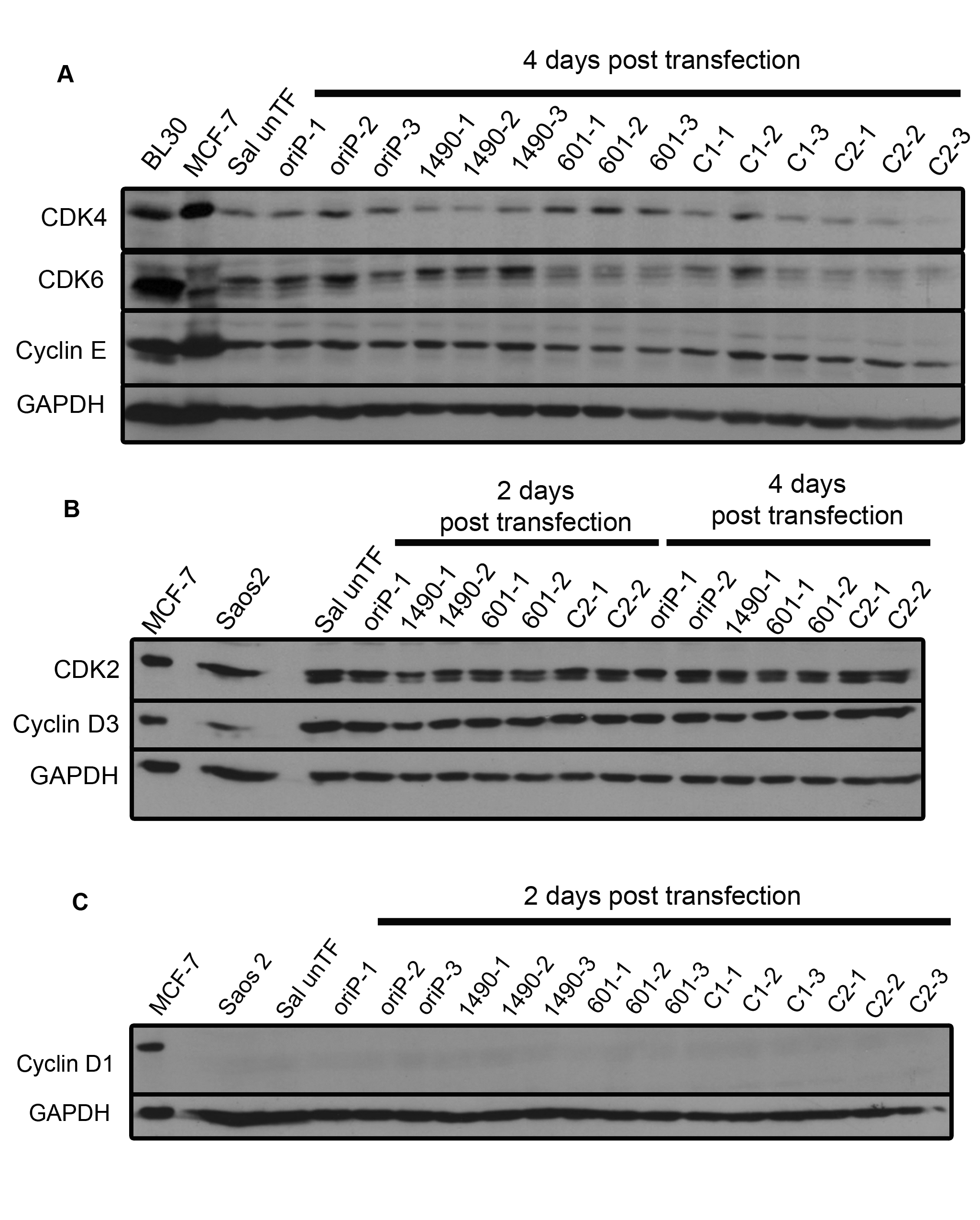

Supplement: Figure S5 — EBNA-3A does not affect expression of G1/S cyclin or CDKs. Immunoblot analysis was performed for (A) CDKs 4, 6, and cyclin E; (B) CDK2 and cyclin D3; and (C) cyclin D1 using lysates from Sal cells transfected with either empty shRNA expression vector (oriP), EBNA-3A-specific (1490 and 601) or control shRNAs (C1 and C2). GAPDH served as a loading control. Representative time points post-transfection are shown, but expression of all proteins was analyzed at 2, 4, and 6 days post-transfection, with no consistent difference between samples, regardless of the level of EBNA-3A. (TIFF) [file ppat.1004415.s005.tiff]

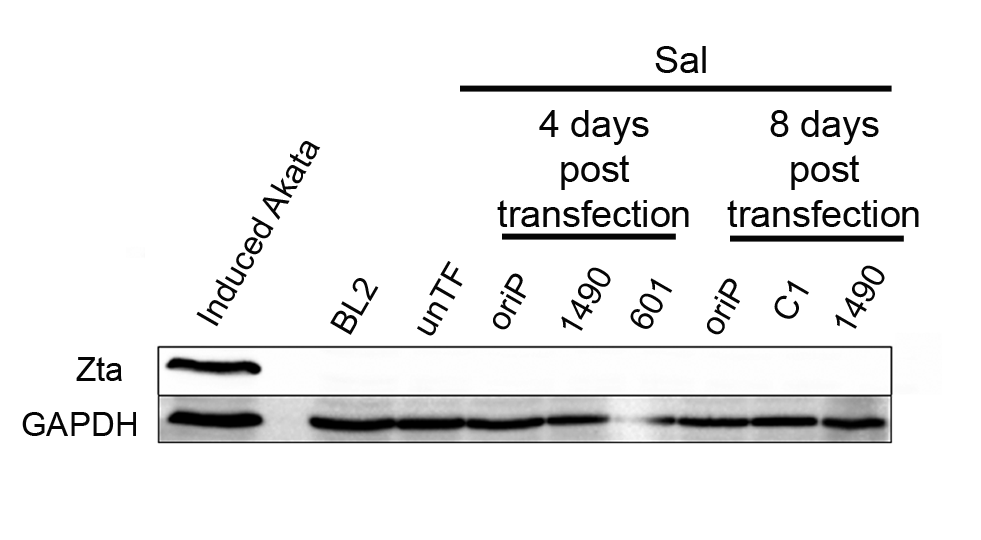

Supplement: Figure S6 — Increased p21 expression following EBNA-3A knockdown is not due to Z expression and lytic reactivation. Sal cells were transfected as previously described and harvested at 4 or 8 days post-transfection in two independent experiments. The productive cycle of replication was induced in EBV-positive Akata cells, which serve as a positive control for Z expression. The EBV-negative BL cell line BL2 serves as a negative control. Immunoblots for Z and GAPDH are shown. (TIF) [file ppat.1004415.s006.tif]

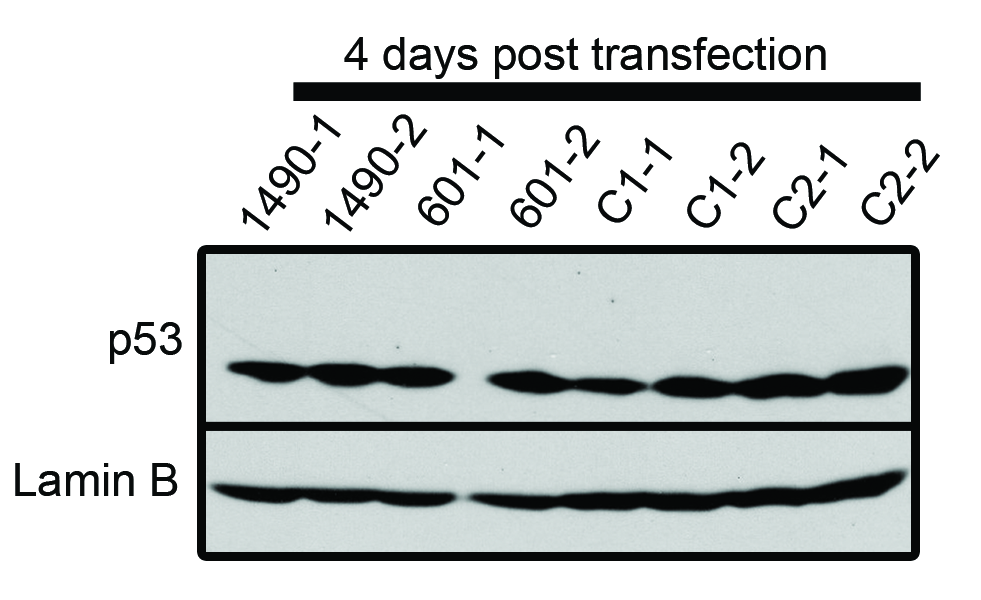

Supplement: Figure S7 — Loss of proliferation in LCLs following EBNA-3A knockdown is not due to elevated p53 expression. MH-LCLs were transfected as described previously, and lysates were harvested at 4 days post-transfection. Immunoblots for p53 and Lamin B are shown. (TIF) [file ppat.1004415.s007.tif]

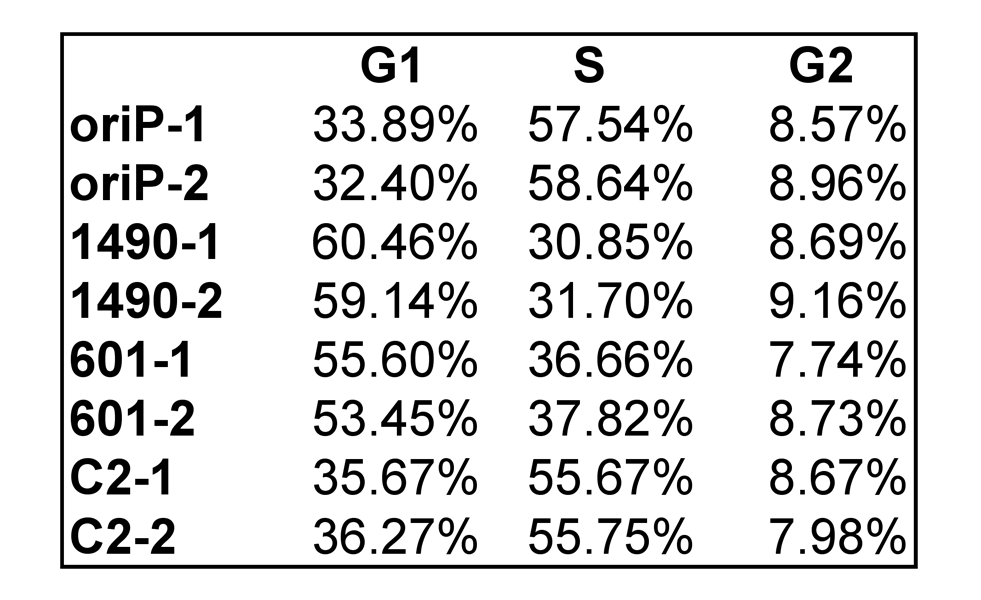

Supplement: Table S1 — Knockdown of EBNA-3A with either shRNA results in G0/G1 cell cycle arrest while control shRNAs have no effect. Sal cells were transfected as described previously and cell cycle analysis was performed as described for Figure 4. (TIF) [file ppat.1004415.s008.tif]
